# Two Different Isomers of Vitamin E Prevent Bone Loss in Postmenopausal Osteoporosis Rat Model

**DOI:** 10.1155/2012/161527

**Published:** 2012-10-15

**Authors:** Norliza Muhammad, Douglas Alwyn Luke, Ahmad Nazrun Shuid, Norazlina Mohamed, Ima-Nirwana Soelaiman

**Affiliations:** ^1^Department of Pharmacology, Faculty of Medicine, Universiti Kebangsaan Malaysia, Jalan Raja Muda Abdul Aziz, 50300 Kuala Lumpur, Malaysia; ^2^Department of Clinical Oral Biology, Faculty of Dentistry, Universiti Kebangsaan Malaysia, 50300 Kuala Lumpur, Malaysia

## Abstract

Postmenopausal osteoporotic bone loss occurs mainly due to cessation of ovarian function, a condition associated with increased free radicals. Vitamin E, a lipid-soluble vitamin, is a potent antioxidant which can scavenge free radicals in the body. In this study, we investigated the effects of alpha-tocopherol and pure tocotrienol on bone microarchitecture and cellular parameters in ovariectomized rats. Three-month-old female Wistar rats were randomly divided into ovariectomized control, sham-operated, and ovariectomized rats treated with either alpha-tocopherol or tocotrienol. Their femurs were taken at the end of the four-week study period for bone histomorphometric analysis. Ovariectomy causes bone loss in the control group as shown by reduction in both trabecular volume (BV/TV) and trabecular number (Tb.N) and an increase in trabecular separation (Tb.S). The increase in osteoclast surface (Oc.S) and osteoblast surface (Ob.S) in ovariectomy indicates an increase in bone turnover rate. Treatment with either alpha-tocopherol or tocotrienol prevents the reduction in BV/TV and Tb.N as well as the increase in Tb.S, while reducing the Oc.S and increasing the Ob.S. In conclusion, the two forms of vitamin E were able to prevent bone loss due to ovariectomy. Both tocotrienol and alpha-tocopherol exert similar effects in preserving bone microarchitecture in estrogen-deficient rat model.

## 1. Introduction

Osteoporosis is a disabling and painful condition whereby bone loss predominates, making the bone highly susceptible to fractures [1]. Osteoporosis takes place when bone resorption by osteoclasts far exceeds bone formation by osteoblasts. Abnormalities in endocrine function and metabolism are the most common causes for osteoporosis. In women, estrogen deficiency due to cessation of ovarian function is an important contributing factor for bone loss with advancing age. Other implicated factors in the pathogenesis of osteoporosis include an increase in osteoclast function, inhibition of osteoblast activity, and imbalance in calcium metabolism [2].

Reactive oxygen species (ROS), the radical forms of oxygen, have been linked to many disease processes including osteoporosis. Excessive accumulation of ROS leads to oxidative stress which in turn will cause cellular damage via peroxidation of lipid membrane, proteins, and nucleic acids. Oxidative stress occurs when the body antioxidant defence fails to overcome the generation of ROS. Recent biochemical and genetic studies have provided the evidence to support the link between osteoporosis and oxidative stress [3–5]. Perhaps the most convincing evidence is the study by Muthusami et al. in a postmenopausal osteoporosis rat model, whereby it is shown that the absence of estrogen causes an increase in lipid peroxidation index with a corresponding reduction in the endogenous antioxidant enzymes [6]. Moreover, free radicals are responsible for causing osteoblast apoptosis and reducing osteoblastogenesis. Hydrogen peroxide, the most stable ROS with the highest oxidative activity, has been reported to be involved in the formation and activation of osteoclasts which precede bone resorption [7].

Antioxidant vitamins can potentially be used to treat and prevent the progress of osteoporosis. At the moment, the approach to osteoporosis management is aimed at preventing fractures from taking place (primary prevention), avoiding further fractures (secondary prevention), stabilizing bone metabolism, and relieving the pain. Nonetheless, not a single agent is able to maintain bone mass and density without exerting undesirable and mostly inconvenient adverse effects. This study was carried out in search for an alternative treatment of osteoporosis using two isoforms of vitamin E. This powerful, lipid-soluble antioxidant vitamin is a collective name for tocochromanols, that is, tocopherols and tocotrienols. A previous study showed that alpha-tocopherol and palm vitamin E (which is rich in tocotrienol) maintained bone mineral density (BMD) in an osteoporosis model [8]. The mechanisms through which vitamin E exerts its effect in preventing bone loss and maintaining BMD are still unclear. In the present study, we report the effects of alpha-tocopherol and tocotrienol on bone microarchitecture in ovariectomized rats, a well-established animal model for postmenopausal osteoporosis.

## 2. Materials and Method

### 2.1. Animals

Three-month-old female Wistar rats weighing 200–250 g were randomly divided into five groups with eight rats in each group. The baseline group was killed at the start of the experiment. Another group of rats was the sham-operated and given olive oil (SHAM) which acted as vehicle. The remaining rats were ovariectomized and treated with vehicle (OVX), tocotrienol (OVX  +  PTT) at a dose of 60 mg/kg body weight or similar doses of alpha-tocopherol (OVX  +  ATF). Treatment commenced two weeks after ovariectomy to allow the rats to recuperate. The olive oil, tocotrienol, or tocopherol was given orally to the rats using an oral gavage needle six days a week for four weeks. Rats were housed in standard cages in groups of three at room temperature with a 12 h light-dark cycle. They were fed with commercial rat chow diet (Gold Coin, Klang, Selangor, Malaysia). Tap water was given *ad libitum*. The study was conducted with the approval from the Universiti Kebangsaan Malaysia Animal Ethics Committee (approval number FAR/IMA/23-JULY/075).

### 2.2. Tocotrienol and Alpha-Tocopherol

Alpha-tocopherol was purchased from Sigma Chemical Company (USA). Pure tocotrienol was prepared from palm oil by the Palm Oil Research Institute of Malaysia (PORIM; Selangor, Malaysia) and had the following composition: 37.2% alpha-tocotrienol, 39.1% gamma-tocotrienol, and 22.6% delta-tocotrienol. The total tocotrienol composition was 98.79%. The analysis of the palm tocotrienol was done using HPLC on Hewlett Packard HP 1100 with 0.5% IPA/Hexane as mobile phase and detected by a fluorescence detector. No alpha-tocopherol detected on HPLC. 

Alpha-tocopherol and tocotrienol were diluted separately in olive oil (Bertolli Classico, Italy) to obtain the concentration of 60 mg/kg body weight. 

### 2.3. Bone Histomorphometry

At necropsy, the femora were taken and fixed in 4% formaldehyde solution for 24 hours before further processing. Undecalcified bone sections from left femurs were prepared according to the procedure as described by Difford [9]. The distal halves of the femurs were cut in saggital plane using a rotary electronic saw (Black & Decker, USA) and then embedded in methyl methacrylate polymer. A heavy duty microtome (Model 2135; Leica, Germany) was used to cut serial bone sections at 8 microns thick. For structural histomorphometry, the undecalcified bone sections were stained with Von Kossa. Histomorphometric measurements were carried out on the secondary spongiosa of the distal femoral metaphysic at distances between 3 mm to 7 mm from the lowest point of the growth plate and from 1 mm of the bilateral cortices. Total tissue area, cancellous bone area, bone surface, and perimeter data were obtained under light microscope (Leica, Germany) at 4x objective magnification with the aid of an image analyzer (VideoTest-Master, Russia). The structural parameters were as follows: trabecular bone volume (BV/TV)—the amount of trabecular bone within the cancellous space, expressed as %—trabecular thickness (Tb.Th)—the mean thickness of trabecular, expressed as *μ*m—trabecular number (Tb.N)—the mean number of trabeculae expressed as per mm—and Trabecular separation (Tb.S)—the distance between two trabecular edges, expressed in *μ*m.

Cellular parameters were obtained from decalcified sections of right femoral bones. The bones were decalcified in EDTA solution for five weeks and then dehydrated in graded concentrations of ethanol before being embedded in paraffin wax. The decalcified femur bones were sectioned at 5 microns thick using a microtome and later the sections were stained with Hematoxylin and Eosin (H&E). The parameters were osteoclast surface (Oc.S) and osteoblast surface (Ob.S). These parameters were calculated as the percentage of the total bone surface as seen under a light microscope (Olympus BX50, USA) interfaced with an image analyzer (Image Pro-Express, Media Cybernetics, USA).

All the formula, nomenclature, symbols, and units used in this study are those recommended by the American Society for Bone and Mineral Research (ASBMR) Nomenclature Committee [10].

## 3. Statistical Analysis

Statistical tests showed that all the data were normally distributed. ANOVA test was carried out followed by Tukey's HSD with *P* < 0.05 considered as significantly different.

## 4. Results

### 4.1. Body Weight

After four weeks of treatment, all groups of rats showed a consistent increase in body weight throughout the study period. However, the ovariectomized rats had a significant increase in body weight at the end of study compared to the sham and treated rats (Table 1).

### 4.2. Bone Histomorphometric Parameters

**Figure 1:**
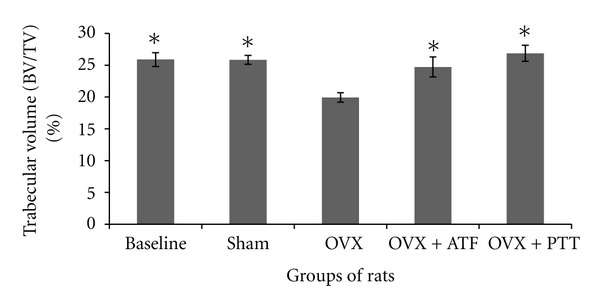
Mean percentage of bone volume to tissue volume. *Indicates significant difference from ovariectomized group (*P* < 0.05). Data are mean ± SEM. OVX: ovariectomy; OVX + ATF: ovariectomy treated with alpha-tocopherol; OVX + PTT: ovariectomy treated with pure tocotrienol.

**Figure 2:**
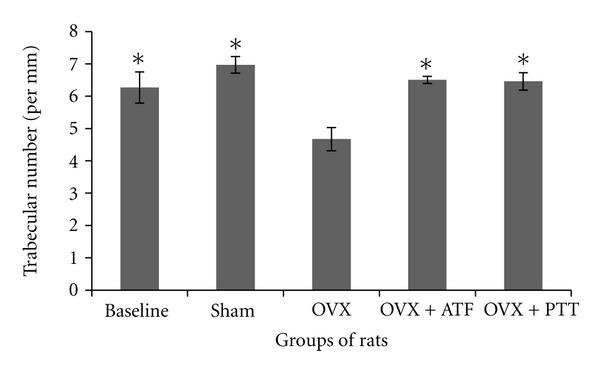
Trabecular number in different groups of rats. *Indicates significant difference from ovariectomized group (*P* < 0.05). Data are mean ± SEM. OVX: ovariectomy; OVX + ATF: ovariectomy treated with alpha-tocopherol; OVX + PTT: ovariectomy treated with pure tocotrienol.

**Figure 3:**
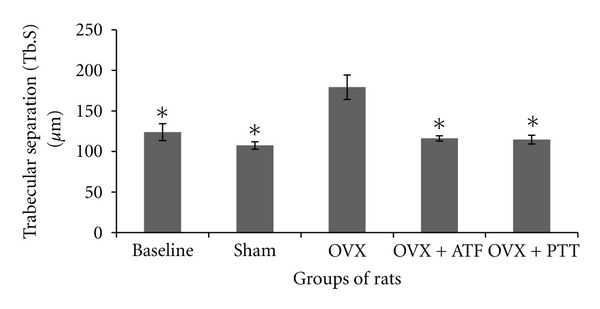
Trabecular separation in different groups of rats. *Indicates significant difference from ovariectomized group (*P* < 0.05). Data are mean ± SEM. OVX: ovariectomy; OVX + ATF: ovariectomy treated with alpha-tocopherol; OVX + PTT: ovariectomy treated with pure tocotrienol.

**Figure 4:**
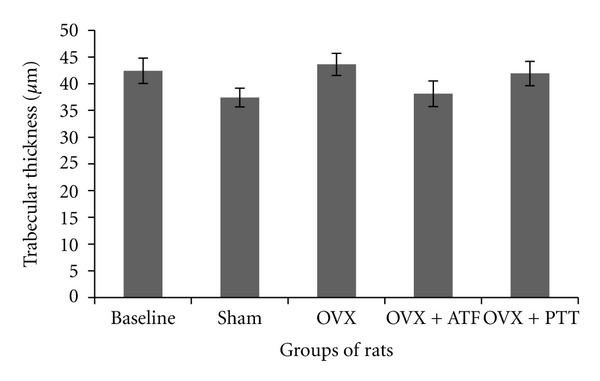
Trabecular thickness in different groups of rats. OVX + PTT: ovariectomy + pure tocotrienol. Data are mean ± SEM. OVX: ovariectomy; OVX + ATF: ovariectomy treated with alpha-tocopherol; OVX + PTT: ovariectomy treated with pure tocotrienol.

Ovariectomized rats had a significantly reduced bone volume (BV/TV) and trabecular number (Tb.N) compared to baseline, sham, and treated groups, while trabecular separation (Tb.S) was increased significantly (Figures 1, 2, and 3). Ovariectomy also caused significant increases in both Osteoclast Surface (Oc.S) and Osteoblast Surface (Ob.S) compared to the other three groups (Table 2). Ovariectomy did not cause any change to trabecular thickness (Tb.Th) parameter (Figure 4).

Treatment of ovariectomized rats with either alpha-tocopherol or tocotrienol prevented the reduction in trabecular bone volume and trabecular number and prevented the increase in trabecular separation. Rats treated with both forms of vitamin E had significantly higher BV/TV and Tb.N while Tb.S was significantly lower than the OVX group (Figures 1, 2, and 3). There were no significant changes seen in trabecular thickness parameter (Figure 4). Treatment with palm tocotrienol or alpha-tocopherol also prevented the increase in osteoclast surface. The rats in OVX + PTT and OVX + ATF groups had significantly lower osteoclast surface than the OVX rats (Table 2). Treatment with either forms of vitamin E did not result in any difference compared to the baseline and sham groups in terms of BV/TV, Tb.N, Tb.S, Tb.Th, and Oc.S. However, ovariectomized rats treated with the two types of vitamin E had high osteoblast surface compared to the rats with intact ovaries (Table 2).

### 4.3. Bone Histology

**Figure 5:**
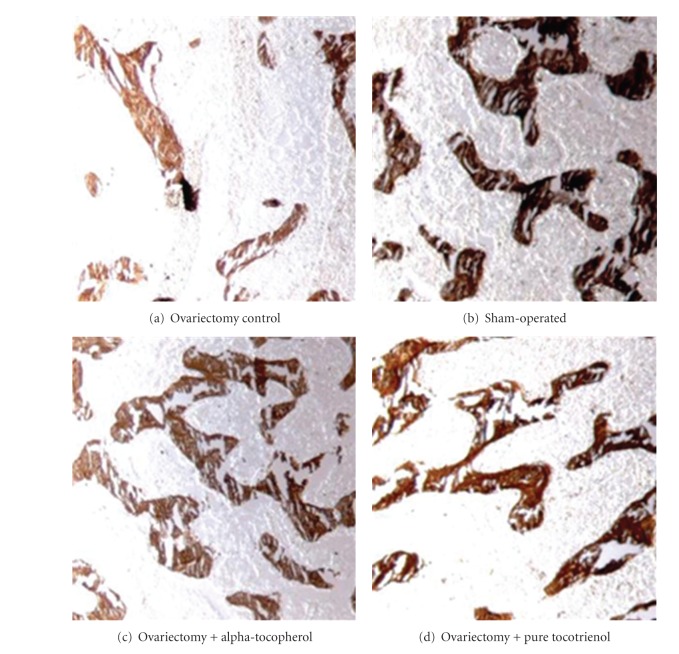
Photomicrographs of distal femur metaphyses from ovariectomized (a) and sham-operated (b) rats, as well as ovariectomized rats treated with alpha-tocopherol (c) and tocotrienol (d). Undecalcified histological bone sections stained with Von Kossa. Trabecular bones appear dark by Von Kossa staining. Loss of trabecular bone is apparent in A while treatment with either forms of Vitamin E prevented bone loss in ovariectomized rats ((c) and (d)). Light microscopy at magnification ×40.

Figure 5 shows photomicrographs of distal femur metaphyses taken from a rat representing each group. Loss of trabecular bone is apparent in those who were ovariectomized (Figure 5(a)) while treatment with either forms of vitamin E prevented bone loss in ovariectomized rats (Figures 5(c) and 5(d)).

## 5. Discussion

The effects of ovariectomy on weight gain have long been established. Ovariectomized rats had increased food intake as their appetite was increased [11, 12]. This change in appetite is partly due to low levels of leptin released by adipose tissue when estrogen is deficient. Leptin works on hypothalamus to control food intake and energy expenditure [13, 14]. 

Removal of ovaries causes osteopenia in rats and ovariectomized animals have been used as a model for postmenopausal bone loss [15–18]. In our study, loss of bone with an increase in resorption and formation indices was seen in ovariectomized rats. Structural changes were evident in these rats whereby their trabecular bone volume was significantly lower than the control groups. Total number of trabecular bones was reduced while the bones were widely separated from one another, as seen in the high value of trabecular separation index. The resorption index which is the osteoclast surface (Oc.S) was increased by two fold compared to the sham rats. Ovariectomized rats also showed an increase in the formation index, the osteoblast surface (Ob.S). The increase in both resorption and formation indices showed an increase in bone turnover rate due to estrogen deficiency. Bone resorption and osteoclastic activities have to be greater than the bone formation by osteoblast in order to account for the net loss of bone. The bone loss is reflected in the photomicrograph of the trabecular bone whereby the bones of the ovariectomized rats showed perforations and discontinued trabeculae compared to the sham-operated group (Figures 5(a) and 5(b), resp.). These findings in postmenopausal rat model are consistent with previous studies [19, 20].

The present study also showed a decrease in trabecular number together with an increase in trabecular surface, along with a reduction in trabecular volume without any changes in trabecular thickness. These observations are consistent with previous reports [12, 21]. They proved that the main mechanism of ovariectomy-induced bone loss is due to perforation and loss of trabecular as a result of osteoclast resorption. The loss of bone is not accompanied by thinning of bone plates. This osteoclastic resorption eventually leads to total loss of trabecular bone. Perforation is the main mechanism of bone loss at the early stage of estrogen deficiency. This is the rapid phase of bone loss which happens only transiently, giving rise to the name “remodelling transient” [20]. The subsequent loss of bone is accompanied by thinning of trabeculae, as seen in long-term studies involving ovariectomized rats [22].

The cellular and molecular mechanisms through which estrogen deficiency stimulates bone resorption are increasingly well understood. Estrogen deficiency upregulates RANKL which leads to an increase in osteoclast recruitment and activation as well as a decrease in osteoclast apoptosis. Lack of estrogen also reduces OPG production by osteoblastic cells causing an increase in the RANKL/OPG ratio that favors bone resorption. In addition, there were reports that estrogen suppresses the expression of bone-resorbing cytokines like M-CSF, TNF-*α*, IL-1, and IL-6 and that lack of estrogen increases these factors. Estrogen also has direct effects on osteoclast and deficiency in this hormone will directly inhibit apoptosis of osteoclast precursor cells and increase osteoclast precursor differentiation into mature osteoclasts [23].

The effects of estrogen deficiency on the skeletal system may be caused by the increase in free radical activities. Recently, there have been a lot of studies that show the link between reactive oxygen species, estrogen deficiency, and bone loss. Several of the intracellular signals essential for osteoclast formation such as nuclear factor-kappa B (NF-〈kappa〉B), c-Jun amino-terminal kinase, and phosphatidylinositol 3-kinase are sensitive to reactive oxygen species [24]. Acute loss of estrogens increases the levels of ROS and activates NF-*κ*B. It also enhances the phosphorylation of p66^shc^, a redox enzyme which amplifies ROS generation and stimulates osteoblast apoptosis [25]. Another study showed that estrogen deficiency lowered antioxidant defences in osteoclasts resulting in increased osteoclastic resorption [7].

Administration of either alpha-tocopherol or pure tocotrienol prevents ovariectomy-induced bone loss. This is evident in the present study by the increase in trabecular bone volume with a corresponding increase in trabecular number together with a decrease in trabecular separation. Photomicrographs of the trabecular bone of rats given alpha-tocopherol and palm tocotrienol appear similar to the sham rats (Figures 5(c) and 5(d)). This result is consistent with another study which also used vitamin E to prevent osteopenia induced by nicotine administration [26]. The bone loss protecting effect by vitamin E also corresponds to the reduction in osteoclast surface with a parallel increase in osteoblast surface as shown in the current study. The findings of the present study strongly indicate that vitamin E may preserve the bone microarchitecture by inhibiting osteoclastogenesis and stimulating osteoblasts to synthesis more bone. The anabolic property of vitamin E has been proven in a previous study whereby it increases bone volume in male rats supplemented with either alpha-tocopherol or tocotrienol [27]. In addition, vitamin E may prevent trabecular bone loss by enhancing bone calcification and mineralization [8, 28].

The primary role of antioxidant vitamins in scavenging the ROS in oxidative stress is already well established. The vitamin E family consists of eight naturally occurring isomers which are *α*-, *β*-, *γ*-, and *δ*-tocopherols as well as *α*-, *β*-, *γ*-, and *δ*- tocotrienols. Most of the studies involving vitamin E reported on the alpha-tocopherol isomer since it is widely available in the market. Tocotrienols are similar to tocopherols except that they have three double bonds in the hydrocarbon tail instead of a saturated tail as found in tocopherols [29, 30]. Our test compound consisted of pure tocotrienols without any tocopherols detected on HPLC. The tocotrienols are made up mainly of the gamma isomer, followed closely by the alpha and the remaining is the delta tocotrienol. The effects of alpha-tocopherol in this study are comparable to those of pure tocotrienols. However, the majority of studies investigating the effects of vitamin E on bone indicated that tocotrienols were better than tocopherols [26, 31–33].

The tocotrienol dose used in the present study was based on the previous studies by Ahmad et al. [33] which showed that, at the dose of 60 mg/kg body weight, tocotrienol was able to prevent the increase of bone-resorbing cytokines in a free-radical-induced rat model. This dose was proven to be safe as toxicity studies in rats showed there was no adverse effect observed even at an extremely high dose of 2500 mg/kg body weight [34]. 

The positive effects of vitamin E on bone by preventing oxidative stress could be mediated via similar pathway involving the RANK/RANKL. Lee et al. [35] and Ha et al. [36] showed that Vitamin E prevented osteoclastogenesis and bone resorption by suppressing RANKL expression and signalling without affecting OPG expression. Vitamin E has also been shown to inhibit the release and expression of bone-resorbing cytokines [36]. These limited studies on the action of vitamin E could suggest that it might exert its effects directly on osteoclast recruitment and osteoclastogenesis. Further studies are of course warranted in order to ascertain its exact mechanism of action on bone metabolism.

In conclusion, supplementation with vitamin E either in the form of alpha-tocopherol or tocotrienol prevented bone loss and maintained the bone microarchitecture in osteopenic rats induced by ovariectomy. Further studies are required to explore the potential of different vitamin E isomers in metabolic bone diseases. 

## Figures and Tables

**Table 1:** Mean body weight.

	Baseline	Sham	Ovx	Ovx + ATF	Ovx + PTT
Week 0	171 ± 0.11	178 ± 0.09	173 ± 0.1	177 ± 0.12	175 ± 0.08
Week 6	—	269 ± 0.12^a^	293 ± 0.11	261 ± 0.13^a^	263 ± 1.12^a^

Data are mean ± S.E.M.

^a^Indicates significant difference compared to ovariectomy (Ovx) group (*P* < 0.05).

Ovx + ATF: ovariectomy + alpha-tocopherol; Ovx + PTT: ovariectomy + pure tocotrienol.

**Table 2:** Cellular parameters.

	Baseline	Sham	Ovx	Ovx + ATF	Ovx + PTT
Oc.S (%)	2.7 ± 0.35^a^	3.08 ± 0.26^a^	7 ± 0.88	3.2 ± 0.35^a^	2.95 ± 0.25^a^
Ob.S (%)	9.9 ± 0.66^a^	9.49 ± 0.38^a^	21.54 ± 1.47	18.15 ± 2.3^b^	19.7 ± 1.12^b^

Data are mean ± S.E.M.

^a^Indicates significant difference compared to ovariectomy (Ovx) group (*P* < 0.05).

^b^Indicates significant difference compared to baseline and sham (*P* < 0.05).

Ovx + ATF: ovariectomy + alpha tocopherol; Ovx + PTT: ovariectomy + pure tocotrienol; Oc.S: osteoclast surface; Ob.S: osteoblast surface.

## References

[1] Doran PM, Khosla S, Hall JE, Nieman LK (2003). Osteoporosis. *Contemporary Endocrinology: Handbook of Diagnostic Endocrinology*.

[2] Parfitt AM, Marcus R, Feldman D, Kelsey J (2000). Skeletal heterogeneity and the purposes of bone remodeling: implications for the understanding of osteoporosis. *Osteoporosis*.

[3] Bax BE, Alam ASMT, Banerji B (1992). Stimulation of osteoclastic bone resorption by hydrogen peroxide. *Biochemical and Biophysical Research Communications*.

[4] Garrett IR, Boyce BF, Oreffo ROC, Bonewald L, Poser J, Mundy GR (1990). Oxygen-derived free radicals stimulate osteoclastic bone resorption in rodent bone in vitro and in vivo. *Journal of Clinical Investigation*.

[5] Dröge W (2002). Free radicals in the physiological control of cell function. *Physiological Reviews*.

[6] Muthusami S, Ramachandran I, Muthusamy B (2005). Ovariectomy induces oxidative stress and impairs bone antioxidant system in adult rats. *Clinica Chimica Acta*.

[7] Lean JM, Davies JT, Fuller K (2003). A crucial role for thiol antioxidants in estrogen-deficiency bone loss. *Journal of Clinical Investigation*.

[8] Norazlina M, Ima-Nirwana S, Gapor MTA, Kadir Khalid BA (2002). Tocotrienols are needed for normal bone calcification in growing female rats. *Asia Pacific Journal of Clinical Nutrition*.

[9] Difford J (1974). A simplified method for the preparation of methyl methacrylate embedding medium for undecalcified bone. *Medical Laboratory Technology*.

[10] Parfitt AM, Drezner MK, Glorieux FH (1987). Bone histomorphometry: standardization of nomenclature, symbols, and units. Report of the ASBMR Histomorphometry Nomenclature Committee. *Journal of Bone and Mineral Research*.

[11] Kippo K, Hannuniemi R, Virtamo T (1995). The effects of clodronate on increased bone turnover and bone loss due to ovariectomy in rats. *Bone*.

[12] Tanizawa T, Yamaguchi A, Uchiyama Y (2000). Reduction in bone formation and elevated bone resorption in ovariectomized rats with special reference to acute inflammation. *Bone*.

[13] Cnop M, Landchild MJ, Vidal J (2002). The concurrent accumulation of intra-abdominal and subcutaneous fat explains the association between insulin resistance and plasma leptin concentrations: distinct metabolic effects of two fat compartments. *Diabetes*.

[14] Mayes JS, Watson GH (2004). Direct effects of sex steroid hormones on adipose tissues and obesity. *Obesity Reviews*.

[15] Kalu DN (1991). The ovariectomized rat model of postmenopausal bone loss. *Bone and Mineral*.

[16] Wronski TJ, Yen CF (1991). The ovariectomized rat as an animal model for postmenopausal bone loss. *Cells and Materials*.

[17] Ima-Nirwana S, Norazlina M, Khalid BAK (1998). Pattern of bone mineral density in growing male and female rats after gonadectomy. *Journal of the ASEAN Federation of Endocrine Society*.

[18] Ayres S, Abplanalp W, Liu JH, Subbiah MTR (1998). Mechanisms involved in the protective effect of estradiol-17*β* on lipid peroxidation and DNA damage. *American Journal of Physiology*.

[19] Frost HM, Jee WSS (1992). On the rat model of human osteopenias and osteoporoses. *Bone and Mineral*.

[20] Sims NA, Morris HA, Moore RJ, Durbridge TC (1996). Increased bone resorption precedes increased bone formation in the ovariectomized rat. *Calcified Tissue International*.

[21] Gal-Moscovici A, Gal M, Popovtzer MM (2005). Treatment of osteoporotic ovariectomized rats with 24,25(OH) 2D3. *European Journal of Clinical Investigation*.

[22] Dempster DW, Birchman R, Xu R, Lindsay R, Shen V (1995). Temporal changes in cancellous bone structure of rats immediately after ovariectomy. *Bone*.

[23] Seeman E (2002). Pathogenesis of bone fragility in women and men. *The Lancet*.

[24] Clarke BL, Khosla S (2010). Physiology of bone loss. *Radiologic Clinics of North America*.

[25] Dröge W (2002). Free radicals in the physiological control of cell function. *Physiological Reviews*.

[26] Almeida M, Han L, Ambrogini E, Bartell SM, Manolagas SC (2010). Oxidative stress stimulates apoptosis and activates NF-*κ*B in osteoblastic cells via a PKC*β*/p66shc signaling cascade: counter regulation by estrogens or androgens. *Molecular Endocrinology*.

[27] Hermizi H, Faizah O, Ima-Nirwana S, Ahmad Nazrun S, Norazlina M (2009). Beneficial effects of tocotrienol and tocopherol on bone histomorphometric parameters in Sprague-Dawley male rats after nicotine cessation. *Calcified Tissue International*.

[28] Shuid AN, Mehat Z, Mohamed N, Muhammad N, Soelaiman IN (2010). Vitamin E exhibits bone anabolic actions in normal male rats. *Journal of Bone and Mineral Metabolism*.

[29] Norazlina M, Chua CW, Ima-Nirwana S (2004). Vitamin E deficiency reduced lumbar bone calcium content in female rats. *Medical Journal of Malaysia*.

[30] Serbinova E, Kagan V, Han D, Packer L (1991). Free radical recycling and intramembrane mobility in the antioxidant properties of alpha-tocopherol and alpha-tocotrienol. *Free Radical Biology and Medicine*.

[31] Kamat JP, Sarma HD, Devasagayam TRA, Nesaretnam K, Basiron Y (1997). Tocotrienols from palm oil as effective inhibitors of protein oxidation and lipid peroxidation in rat liver microsomes. *Molecular and Cellular Biochemistry*.

[32] Maniam S, Mohamed N, Shuid AN, Soelaiman IN (2008). Palm tocotrienol exerted better antioxidant activities in bone than *α*-tocopherol. *Basic and Clinical Pharmacology and Toxicology*.

[33] Ahmad NS, Khalid BAK, Luke DA, Nirwana SI (2005). Tocotrienol offers better protection than tocopherol from free radical-induced damage of rat bone. *Clinical and Experimental Pharmacology and Physiology*.

[34] Nakamura H, Furukawa F, Nishikawa A (2001). Oral toxicity of a tocotrienol preparation in rats. *Food and Chemical Toxicology*.

[35] Lee JH, Kim HN, Yang D (2009). Trolox prevents osteoclastogenesis by suppressing RANKL expression and signaling. *Journal of Biological Chemistry*.

[36] Ha H, Lee JH, Kim HN, Lee ZH (2011). *α*-Tocotrienol inhibits osteoclastic bone resorption by suppressing RANKL expression and signaling and bone resorbing activity. *Biochemical and Biophysical Research Communications*.

